# Double Magnet Ingestion Causing Intestinal Perforation with Peritonitis: Case Report and Review of the Literature

**DOI:** 10.1155/2022/4348787

**Published:** 2022-01-15

**Authors:** Yousef S. Abuzneid, Hussam I. A. Alzeerelhouseini, Abdelrahman Rabee, Wafa Aqel, Rawan F. Ayyad, Thikrayat M. Asad, Radwan Abukarsh

**Affiliations:** ^1^Al-Quds University Faculty of Medicine, Jerusalem, State of Palestine; ^2^Palestine Red Crescent Society Hospital, Hebron, State of Palestine

## Abstract

**Introduction:**

Foreign body ingestion is a common pediatric complain, and most can be passed spontaneously; however, magnetic object ingestion is rather rare, and they can cause severe complications when multiple magnets are ingested, as they lead to entrapment of bowel walls between them, causing ischemia, pressure necrosis, perforation, and fistula formation. *Case Presentation*. Herein, we present a case of a 16-month-old female patient presented to our department complaining of continuous vomiting for two days along with fever and irritability. X-ray revealed dilated bowel loops with a radioopaque foreign body in the right lower quadrant. After discussing with the parents, exploratory laparotomy was done, showing two bowel perforations at the site of the magnets. Affected bowel was resected with anastomosis. The patient was discharged after 3 days with an uneventful recovery. *Discussion*. The diagnosis and management of magnet ingestion differ from those of small foreign bodies, which are usually managed conservatively by watchful waiting. Usually, the diagnosis is done due to complications such as peritonitis and death. On the other hand, management depends on the number, size, magnetic field, and shape of the magnet, and whether it has passed the pylorus or not.

**Conclusion:**

It is important to establish the diagnosis of this condition as early as possible to prevent complications. Despite the efforts that were made to try to prevent and minimize the risk of magnet ingestion, more investigations are required to reach a common and united strategy for management of such conditions.

## 1. Introduction

Foreign body ingestion in children is a common condition, particularly in those aged between 6 months and 3 years [1]. As most ingested objects are small, they pass spontaneously, and it is estimated that 40% of foreign body ingestions go unnoticed without showing any signs and symptoms [2]. Once the foreign body reaches the small intestine, in 80-90% of cases, the object passes spontaneously; however, some cases may be complicated by intestinal obstruction, volvulus, intussusception, and perforation [3].

Magnetic object ingestion is rare; however, it is important to be considered and recognized by physicians. Its incidence is expected to be 3.06 cases per 100000 children per year but during the last decade this number has grown fivefold owing to the growing popularity of magnetic toys [4]. A single magnet is expected to behave like other foreign bodies; however, the harm risk is escalated when more than one magnetic object is swallowed and passed beyond the stomach since the pieces might hold the bowel wall in between them resulting in ischemia, pressure necrosis, perforation, and fistula formation [5].

When there is evidence of multiple magnetic foreign body ingestion, our patients should be managed aggressively to prevent complications. Moreover, a midline laparotomy should be used as the incision of choice to facilitate access [3].

## 2. Case Presentation

A 16-month-old female patient was admitted to our pediatric ward due to vomiting for two days. She was doing well until two days prior to admission when she started to vomit. This vomiting was consistent with normal gastric content and happening five to six times with a large content and after each feeding. The next day, she developed fever recorded as 38.3°C and continued vomiting, becoming hypoactive and irritable with continuous crying and inability to sleep. The last two episodes of vomiting were characteristic by a yellowish to greenish content. There was no history of diarrhea, cough, or upper respiratory infection or loss of consciousness. On physical examination, the patient looked ill with flexed lower limbs and dry mucous membranes. The abdomen was moderately distended, tender, and rigid consistent with peritonitis.

**Figure 1:**
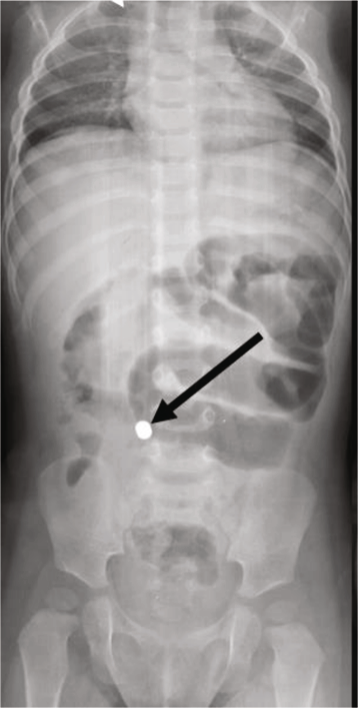
Dilated bowel loops with a radioopaque foreign body (indicated with an arrow).

CBC and blood gas were performed, showing that the hemoglobin was 10.1 g/dL, the MCV was 55 fL, WBCs were 16.7 × 10^9^/L, platelets were 506 × 10^9^/L, the pH was 7.4, the CO_2_ was 35 mmHg, the pO_2_ was 44, and the HCO_3_ was 22.7 mEq/L. Electrolytes were normal, and we did a plain abdominal X-ray (Figure 1) which showed dilated bowel loops with a radioopaque foreign body in the right lower quadrant (mostly representing a swallowed magnet).

A discussion with the family about the possibility of foreign body ingestion was executed but the parents did not know the answer, so a decision of exploratory laparotomy was made and, furthermore, proceeded to do it. On exploration and upon entry to the peritoneal cavity, a gush of pus came out immediately and was suctioned out. Fibrin deposits were also seen between the small bowel loops.

**Figure 2:**
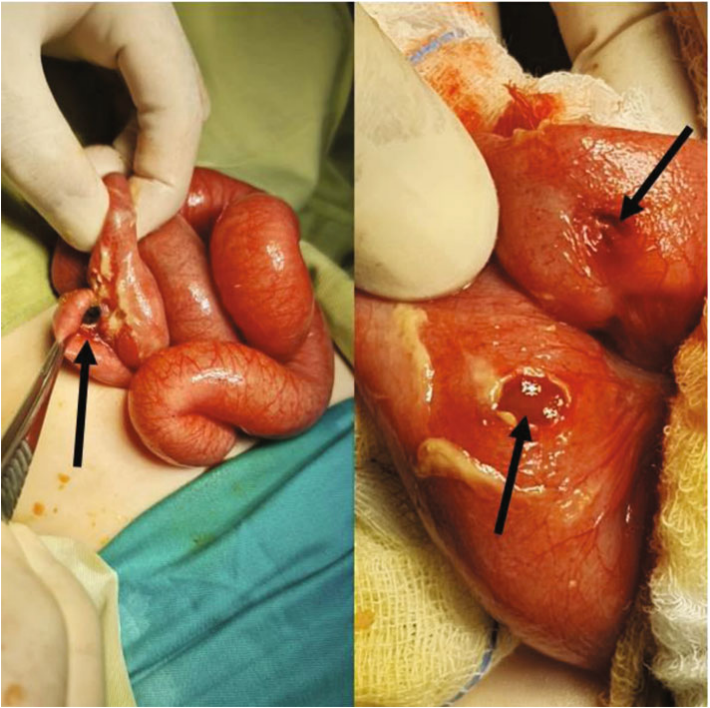
Intestinal perforations (indicated with arrows) as a result of magnet ingestion showing the pus and both perforations.

A foreign body was noticed (two magnets that were stuck together) in the intestinal lumen with a small perforation in the adjacent bowel loop. While removing the magnet, another perforation was noticed underneath, giving as the suspicion that one of the magnets perforated one loop in order to meet with the other magnet (Figures 2). We removed both magnets at the same time because they were stuck together, and we could not separate them.

A segmental resection of 10 cm of the ileum containing both perforated sites with primary anastomosis was executed, and then, the abdomen was closed without the need of a drain insertion, so the patient was sent to the ICU for postoperation follow-up. After few hours, the patient was conscious and feeling better. She stayed in the hospital for three days on IV antibiotics, started feeding on the 2^nd^ day (tolerated oral intake well with smooth post-operative course), and had no complications and an uneventful recovery.

## 3. Discussion

Many of the small foreign bodies that are ingested are managed conservatively by watchful waiting and serial X-ray images; however, in cases of multiple ingestion, X-ray imaging may not be very helpful as magnets can be mistaken for other less harmful inorganic foreign bodies (e.g., pearl) or may be indistinguishable from other metallic foreign bodies (e.g., coins and parts of jewelry) [6, 7]. Moreover, it is difficult to differentiate between a magnetic foreign body and a metallic one just by X-ray imaging [8]. Usually, the diagnosis of magnet ingestion is made due to complications such as peritonitis or death [2, 9]. These cases are difficult to diagnose due to the similarity of their symptoms with those of a typical flu or other gastrointestinal illnesses [10]. Some cases report that most abdominal symptoms occur between one and seven days after the ingestion of multiple magnets [11].

In our patient, we can see that she manifested symptoms, but those symptoms were nonspecific, being fever, hypoactivity, vomiting, irritability, and crying. She also had abdominal distention; however, all these symptoms were a vague hint of the diagnosis, and at the beginning, we did not have any clue about why the patient had those manifestations.

Recent literature suggests that the ingestion of multiple metallic foreign bodies should be considered potentially harmful as alkali batteries because of their ability to split adjacent sections of bowel causing pressure necrosis and subsequent perforation [3, 12, 13]. In some cases of single magnet ingestion, conservative treatment may be sufficient, but magnets can still cause dangerous morbidities depending on the size, shape, and magnetic field; therefore, endoscopic or surgical intervention is occasionally required. However, if more than one magnet is ingested, endoscopic removal must be performed without delay unless the magnets have travelled beyond the pylorus. If the magnets have passed the pylorus, surgical intervention, even if the patient is asymptomatic, is the preferred type of management for some authors to assess the vitality of the bowels [11].

In our case, we did not wait for conservative measures because the child already had features of frank peritonitis. Herein, we decided to perform a laparotomy surgery to visualize all the bowels and asses their viability. During the procedure, we saw that there were two small bowel perforations, and we were able to manage the situation by resecting the affected part of the bowel (10 cm) and doing an anastomosis. After that, the patient had a full recovery and did not have any complication.

Kramer et al. [14] proposed a management algorithm for magnet ingestion in children that we found very appropriate and which every physician should take into consideration in which is recommended what to do if the child ingested one magnet (observation mainly and serial X-rays as outpatient to confirm the passage of the magnet) or if the child ingested two or more magnets (in which the option of surgery is more present due to the possible complications of the magnets depending if they are above or below the stomach).

We performed the surgery in our patient mainly because she presented with signs and symptoms of the complications of magnet ingestion (peritonitis and possible sepsis) and because of the lack of stipulated management for the patient's condition.

With this case report, we empathize the importance of an adequate history taking and diagnosis, and also, we discuss the possible types of management that are recorded in the literature review for this condition, depending on the location, the quantity of the magnets ingested, and the signs and symptoms presented by the patient which can be fatal due to the possible complications that exist.

## 4. Conclusion

Magnet ingestion is considered a rare condition as well as challenging to diagnose before complications of intestinal perforation or peritonitis occur. So, a high index of suspicion is necessary in patients presenting with unexplained gastrointestinal symptoms, and early removal is warranted in cases of multiple magnetic foreign body ingestion to reduce potential morbidity and mortality.

There are not many cases about this topic according to our research and in that many details as the one that we are writing. To our consideration, our case report brings a complete view of all the possible complications and types of management that physicians should know when facing this condition.

## Data Availability

The data supporting this case report are from previously reported studies and datasets, which have been cited.
